# NOD *Scid* Gamma Mice Are Permissive to Allogeneic HSC Transplantation without Prior Conditioning

**DOI:** 10.3390/ijms17111850

**Published:** 2016-11-07

**Authors:** Tom Verbiest, Rosemary Finnon, Natalie Brown, Paul Finnon, Simon Bouffler, Christophe Badie

**Affiliations:** 1 Cancer Mechanisms and Biomarkers Group, Radiation Effects Department, Centre for Radiation, Chemical and Environmental Hazards, Public Health England, Didcot OX11 ORQ, UK; tom.verbiest@oncology.ox.ac.uk (T.V.); rosemary.finnon@phe.gov.uk (R.F.); natalie.brown@phe.gov.uk (N.B.); paul.finnon@phe.gov.uk (P.F.); simon.bouffler@phe.gov.uk (S.B.); 2 CRUK & MRC Oxford Institute for Radiation Oncology, Department of Oncology, University of Oxford, Oxford OX3 7DQ, UK

**Keywords:** hematopoietic stem cells, transplantation, NOD *scid* gamma (NSG), engraftment, hematopoietic niche occupancy defect

## Abstract

*Scid* hematopoietic stem cells (HSCs) have an intrinsic defect in their maintenance within the bone marrow (BM) niche which facilitates HSC transplantation without the absolute requirement of prior conditioning. Nevertheless, NOD *scid* mice have a significantly altered life span due to early development of thymic lymphomas, which compromises the ability to study the long-term fate of exogenous HSCs and their progeny. Here, we present data on the transplantation of HSCs into NOD *scid* gamma (NSG) mice to achieve long-term engraftment without prior conditioning. We transplanted allogeneic HSCs constitutively expressing the mCherry fluorescent marker into age-matched NSG mice and assessed donor chimerism 6 months post-transplantation. All transplanted NSG mice showed long-term myeloid and lymphoid cell chimerism. Also, in vivo irradiated HSCs showed long-term engraftment, although overall white blood cell (WBC) donor chimerism was lower compared with non-irradiated HSCs. Using this novel NSG transplantation model, we will be able to study the effects of low dose in vivo X-ray exposure on the long-term fate of HSCs, without the requirement of prior radio-ablation of the recipient, and thus leaving the recipient’s BM microenvironment uncompromised. In conclusion, we demonstrated for the first time that allogeneic HSCs from a different inbred strain can compete for niches in the BM compartment of NSG mice.

## 1. Introduction

Murine models are indispensable in studying radiation-induced acute myeloid leukemogenesis with no in vitro system satisfactorily mimicking this process. The CBA mouse is the most well characterized model, with close histopathological similarity to that of the human disease [1,2]. Bone marrow (BM) transplantation assays have been extensively used to study molecular and chromosomal processes in the hematopoietic system at various points post-irradiation, and are the gold standard to study hematopoietic stem cells (HSCs) and their long-term engraftment potential [3]. However, one of the major limitations is the requirement of prior conditioning of the recipients (radio-ablation of the BM), where host mice are exposed to lethal doses of X-irradiation [4]. This is a high risk procedure with a number of drawbacks (including gastrointestinal syndrome and BM failure) not least of which is the profound alteration of the host’s BM microenvironment [5].

A variety of epidemiological studies of human cohorts exposed to low dose ionizing radiation provided clear evidence for a direct association between low dose radiation exposure and leukemia incidence. Firstly, it was reported that the overall cancer incidence was 24% greater for people exposed to computed tomography scans in their childhood than for unexposed children. The incidence increased significantly for leukemia and many types of solid cancer [6]. Another study into low dose medical radiation exposure reported that computed tomography scans in children delivering cumulative doses of approximately 50 mGy almost tripled the risk of developing leukemia later in life [7]. Similarly, positive associations between protracted low dose radiation exposure and leukemia have been reported for the Techa River Cohort [8] and radiation-monitored workers [9]. Interestingly, a similar association has been postulated for human cohorts born in areas with high natural background radiation [10,11,12]. The profound changes to the HSC niche following myeloablative conditioning of murine recipients greatly compromise our ability to study low dose radiation exposure and the effects on the HSCs and their contribution towards long-term hematopoiesis in a biologically relevant setting.

Recently, it was reported that an intrinsic defect in the maintenance of *scid* HSCs, the so-called “BM hematopoietic niche occupancy defect”, facilitates exogenous HSC transplantation without prior conditioning [13]. Nevertheless, NOD *scid* mice have a significantly altered life span due to the early development of thymic lymphomas, which compromises the ability to study the long-term fate of exogenous HSCs and their progeny in these recipient mice [14].

NOD *scid* gamma (NOD.Cg-*Prkdc*^scid^ *Il2rg*^tm1Wjl^/SzJ, NSG) mice are highly immunodeficient mice generated by crossing NOD *scid* mice with mice bearing the X-linked B6.129S4-*Il2rg*^tm1Wjl^/J allele: the NOD background contributes to innate immune deficiencies, the *scid* mutation prevents the maturation of lymphocytes and the *Il2rg* deficiency blocks natural killer cell development [15]. Interestingly, NSG mice are resistant to thymic lymphoma development, even following sublethal irradiation [16]. Therefore, we investigated the ability of allogeneic HSCs to achieve long-term engraftment in NSG mice without prior conditioning, and thus leaving the recipient’s BM microenvironment uncompromised.

## 2. Results

### 2.1. CBA/H^mCherry^ Mice Constitutively Express mCherry across HSCs and Downstream Hematopoietic Lineages

**Figure 1:**
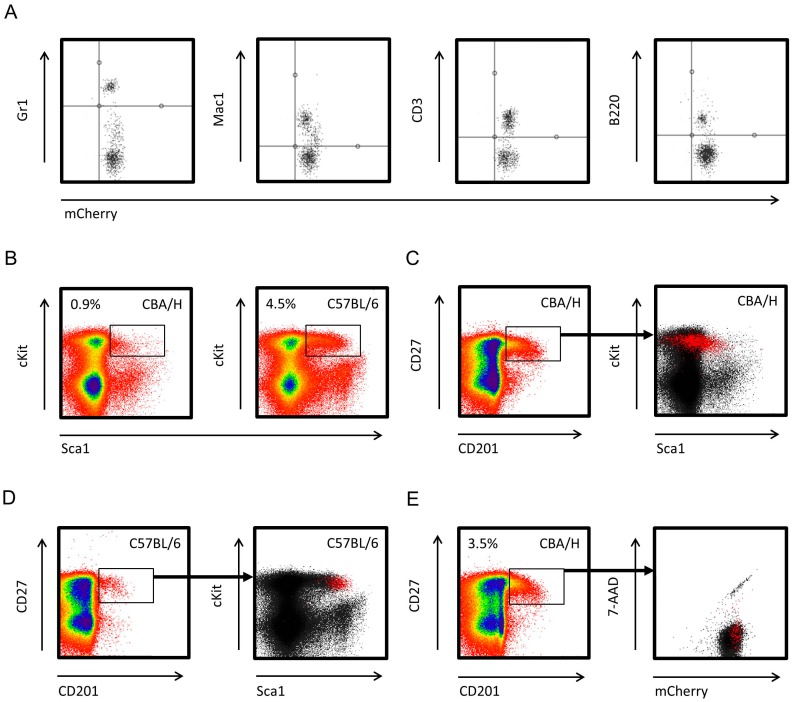
CBA/H^mCherry^ mouse model characterization. Flow cytometry profiles of CBA/H^mCherry^ donor cells, (**A**) mCherry is constitutively expressed across all major types of white blood cells (WBCs). White blood cell single cell suspensions were prepared from 8 weeks old CBA/H^mCherry^ mice (*n* = 3), and single-labelled with fluorescein isothiocyanate (FITC)-tagged antibodies (Gr1-FITC for granulocytes, Mac1-FITC for monocytes, CD3-FITC for T-cells and B220-FITC for B-cells); (**B**) the Lin−/Sca1+/cKit+ population in CBA/H^mCherry^ mice is five-fold smaller compared with C57BL/6 mice (0.9% and 4.5% of Lin− cells are LSK, respectively). BM cells were collected from male 8 weeks old CBA/H^mCherry^ mice (*n* = 3), followed by immunomagnetic isolation of Lin− cells and labelling with antibodies (Sca1-PE-Cyanine7 and cKit-allophycocyanin-eFluor^®^780); (**C**,**D**) the CD201+/CD27+ population partially or completely overlaps the Sca1+/cKit+ population; and (**E**) CBA/H^mCherry^ HSCs (CD201+/CD27+ Lin− cells) constitutively express mCherry.

The tracking of allogeneic cells in NSG recipients required the presence of a marker allowing their detection; here we engineered a mouse model with constitutively active mCherry across all hematopoietic cells (CBA/H^mCherry^ mice were established by microinjection of a Zinc Finger Nuclease pair targeting the insertion site on chromosome 2, together with the Rosa26 promotor and the *mCherry* gene, into the nucleus of CBA/H one-cell embryos). In the peripheral blood of CBA/H^mCherry^ mice, all myeloid (Mac1+ and Gr1+) and lymphoid (CD3+ and B220+) white blood cells (WBCs) expressed mCherry (Figure 1A; WBCs were single-labelled with fluorescein isothiocyanate (FITC)-tagged antibodies).

Various murine strains, including CBA/H and BALB/c mice, express low Sca1 levels on the hematopoietic cells [17]. In CBA/H^mCherry^ mice, the Lin−/Sca1+/cKit+ (LSK) BM population was five-fold smaller compared with age-matched C57BL/6 mice (0.9% and 4.5% of Lin− cells are LSK, respectively; Figure 1B). A recent study demonstrated that labelling Lin− cells with CD201/CD27 antibodies, rather than Sca1/cKit antibodies, allows a more efficient isolation of long-term HSCs (LT-HSCs) in murine strains with phenotypically low Sca1 expression [18]. The CD201+/CD27+ population partially or completely overlapped the Sca1+/cKit+ population (Figure 1C,D). By isolating CD201+/CD27+ cells, the CBA/H^mCherry^ HSC yield increased approximately 4-fold compared with Sca1+/cKit+ gating (3.5% vs. 0.9%, respectively; Figure 1E left panel). The CD201+/CD27+ population is considered to contain the vast majority of LT-HSCs [18], and all these cells expressed mCherry (CD201+/CD27+ Lin− cells in red; Figure 1E right panel). These results demonstrated that CBA/H^mCherry^ HSCs and their myeloid and lymphoid progeny retain mCherry expression throughout their life span, making it a suitable model for tracking donor cells in NSG recipients.

### 2.2. NOD Scid Gamma Bone Marrow (BM) Microenvironment Supports Allogeneic Hematopoietic Stem Cell (HSC) Engraftment and Differentiation

**Figure 2:**
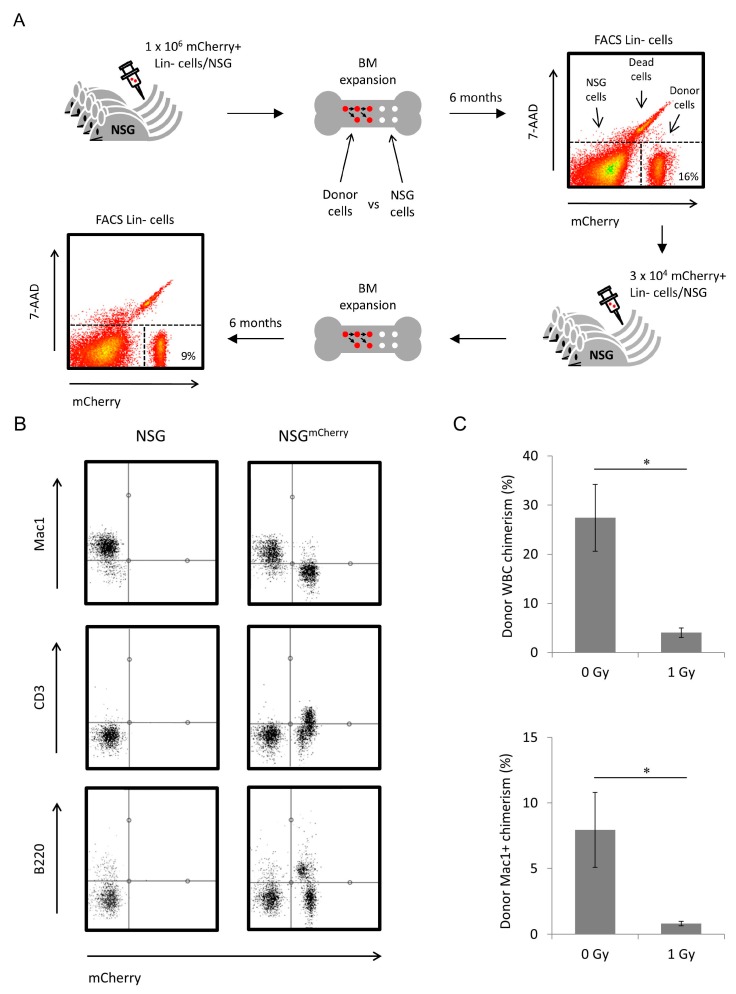
Nonmyeloablated NSG mice are permissive to allogeneic HSC transplantation. (**A**) Six months following tail vein injection of Lin− cells, NSG recipients (*n* = 5) were sacrificed, followed by immunomagnetic isolation of Lin− cells and analysis of donor contribution in the BM. mCherry+ Lin− cells were reinjected into secondary male NSG recipients (*n* = 5). Six months later, donor contribution in the peripheral blood and BM was analyzed; (**B**) representative multilineage NSG blood reconstitution (B-cells, B220+/mCherry+; T-cells, CD3+/mCherry+; monocytes and granulocytes, Mac1+/mCherry+); and (**C**) donor chimerism in peripheral blood, comparing non-irradiated and 1 Gy irradiated CBA/H^mCherry^ Lin− cells. Donor blood chimerism of Mac1 is the Mac1+/mCherry+ WBCs of the total Mac1+ WBC population. Error bars indicate SEM and significance was determined by Student *t* test; * *p* < 0.05.

Considering *scid* HSCs have an intrinsic defect in their maintenance within the BM niche, we next investigated whether transplantation of CBA/H^mCherry^ Lin− cells could achieve long-term HSC engraftment in NSG mice without prior conditioning. Femurs, tibias, iliac crests and spines from male adult (8 weeks old) CBA/H^mCherry^ mice were crushed, and negatively selected for Lin− cells, hereby depleting the BM of mature, lineage-committed cells. Five age-matched male NSG mice were tail vein injected with 1 × 10^6^ Lin− cells in 150 μL IMDM (containing approximately 1 × 10^4^ CD201+/CD27+ HSCs; Figure 2A).

Six months following primary transplantation, all transplanted NSG mice presented with donor-derived lymphoid cells in the peripheral blood, both B-cells (B220+/mCherry+) and T-cells (CD3+/mCherry+), in contrast to the peripheral blood of NSG controls, which lacked B-cells (B220−) and T-cells (CD3−) (Figure 2B right and left panels, respectively). In addition, all recipients showed myeloid cell chimerism (approximately 8% of granulocytes and monocytes in the recipients’ blood were donor-derived, Mac1+/mCherry+; Figure 2C), and 16% of Lin− cells in the recipients’ BM were donor-derived (Figure 2A).

Subsequently, we isolated mCherry+ Lin− cells from the primary NSG recipients’ BM and reinjected these cells into five secondary NSG recipients (3 × 10^4^ mCherry+ Lin− cells/secondary NSG mouse; Figure 2A). Six months following secondary transplantation, approximately 9% of Lin− cells isolated from secondary NSG recipients were donor-derived (mCherry+; Figure 2A). Lin- cells were then stained with CD201/CD27 antibodies to identify transplanted HSCs (mCherry+/CD201+/CD27+ Lin− cells) and peripheral blood analysis of secondary recipients showed multilineage reconstitution.

Although NSG mice have a similar intrinsic BM hematopoietic niche occupancy defect as NOD *scid* mice, we hypothesized that the reduced “fitness” of irradiated donor HSCs might be a limiting factor towards the success of this novel transplantation model. Therefore, to determine the ability of irradiated HSCs to achieve long-term engraftment in NSG mice, we exposed CBA/H^mCherry^ mice to 1 Gy X-rays 7 days prior to BM harvest, isolation and intravenous injection of 1 × 10^6^ Lin− cells into five age-matched male NSG recipients. Six months following transplantation, all NSG mice showed long-term multilineage engraftment, although overall WBC donor chimerism was lower compared with non-irradiated Lin− cells; Figure 2C; *p* < 0.05.

## 3. Discussion

Our findings indicate that CBA/H^mCherry^ HSCs can compete for niches in the nonmyeloablated NSG BM compartment and that the nonmyeloablated NSG BM microenvironment is capable of supporting allogeneic long-term HSC engraftment and differentiation. The percentage of CBA/H^mCherry^ cell chimerism in the blood of adult nonmyeloablated NSG mice 6 months following engraftment of 1 × 10^6^ CBA/H^mCherry^ Lin− cells was similar to the cell chimerism level reported in the blood of nonmyeloablated NSG mice 14 weeks following transplantation of 1 × 10^6^ wild-type total bone marrow cells. Interestingly, the percentage of CBA/H^mCherry^ cell chimerism in the bone marrow of adult nonmyeloablated NSG mice was higher than reported in that study (16% and 6%, respectively). This difference could be explained by the fact that we used Lin− cells, which represent an enrichment of about fifty fold in terms of progenitors and stem cells compared with total bone marrow. Andrade and colleagues observed a significant increase in peripheral blood and bone marrow chimerism with prior sublethal total body irradiation (2.7 Gy) of the recipients [19]. This has also been confirmed in our laboratory with 2.5 Gy total body irradiated NSG recipient mice 24 h before injection of donor cells (unpublished data). The peripheral blood chimerism level we observed in NSG mice transplanted with sham-irradiated cells is in agreement with those previously reported following transplantation of 5 × 10^6^ wild-type total bone marrow cells in unconditioned *scid* mice (12%–18% of myeloid cells and 8%–10% of LSK cells) [13].

Several recent publications have studied human HSC engraftment in immunodeficient mice. The percentage of CBA/H^mCherry^ cell chimerism in the blood of adult nonmyeloablated NSG mice 6 months following engraftment of 1 × 10^4^ CBA/H^mCherry^ CD201+/CD27+ HSCs (27%) was similar to the human cell chimerism level reported in the blood of 2.5 Gy myeloablated NSG mice 8 weeks following engraftment of 5 × 10^4^ CD34+ human HSCs (22%) [20]. Our observed cell chimerism was higher compared with the human cell chimerism level reported in blood of newborn nonmyeloablated NSG mice 12 weeks following engraftment of 3 × 10^4^ CD34+ human HSCs, but lower compared with the human cell chimerism level reported in blood of adult nonmyeloablated NSG mice 12 weeks following engraftment of 3 × 10^4^ CD34+ human HSCs (18% and 41%, respectively) [21]. The authors did not observe a statistical difference in human cell chimerism levels in the blood of nonmyeloablated NSG mice compared with newborn or adult 1 Gy myeloablated NSG mice [21]. A more recent study reported a much lower human cell chimerism level in blood of adult nonmyeloablated NSG mice 12 weeks following engraftment of 2.5 × 10^5^ CD34+ human HSCs (8%). In contrast with the previous studies, the authors did observe a significantly enhanced human cell chimerism level in the blood of 2.5 Gy myeloablated NSG mice compared with nonmyeloablated NSG mice (approximately 60% and 8%, respectively) [22].

Using this novel NSG transplantation model, we will be able to study the effects of low dose in vivo X-ray exposure on the long-term fate of HSCs, without the requirement of prior radio-ablation of the recipient, and thus leaving the recipient’s BM microenvironment uncompromised. In addition, titration studies or competitive studies between strains could provide further insightful information. In conclusion, we demonstrated for the first time that allogeneic HSCs from a different inbred strain can compete for niches in the BM compartment of nonmyeloablated NSG mice, allowing allogeneic HSC engraftment and multilineage reconstitution.

## 4. Materials and Methods

### 4.1. Mice

A chr2 Rosa26-mCherry mouse model (CBA/H^mCherry^) was established by microinjection of a Zinc Finger Nuclease pair targeting the insertion site on chromosome 2, together with the Rosa26 promotor and the *mCherry* gene, into the nucleus of CBA/H one-cell embryos. CBA/H^mCherry^ male mice were used for experimental purposes at 10 weeks old. Male 8 weeks old NSG mice were housed in containment isolators and habituated for 2 weeks prior to experimental use. Mice were provided with sterile water and food ad libitum, subjected to a 12 h light/ 12 h dark cycle. All animal procedures conformed to the United Kingdom Animals (Scientific Procedures) Act 1986, Amendment Regulations 2012. Experimental protocols were approved by the Home Office and institutional AWERB (PPL 30/3355; 21 December 2015).

### 4.2. Long-Term Transplantation Assays

CBA/H^mCherry^ mice were sham-irradiated or 1 Gy irradiated 7 days prior to harvest, using an X-ray source (AGO, West Coker, UK) with a dose rate of 0.5 Gy/min (250 kVp and 13 mA). Femurs, tibias, iliac crests and spine were removed and crushed, followed by immunomagnetic isolation of Lin− cells (EasySep^TM^, Stem Cell Technologies, Grenoble, France; using manufacturer’s protocol). One million Lin− cells were resuspended in 150 μL IMDM (Sigma, Dorset, UK) before injection via the tail vein of age-matched NSG recipients.

### 4.3. Flow Cytometry

Following red blood cell lysis, white blood cells (WBCs) were single-labelled with antibodies (CD3-fluorescein isothiocyanate (FITC), B220-FITC, Gr1-FITC and Mac1-FITC; BD Biosciences, London, UK), and washed twice with PBS before analysis on a Guava easyCyte^TM^ (Millipore, Watford, UK). Lin− cells were labelled with antibodies (CD27-FITC, CD201-phycoerythrin (PE), Sca1-PE-Cyanine7 and cKit-allophycocyanin-eFluor^®^780 (Biolegend, London, UK)) and analyzed on a MoFlo™ XDP (Beckman Coulter, High Wycombe, UK). To exclude dead cells, 7-Amino-Actinomycin D (7-AAD; BD Pharmigen, Oxford, UK) was added 10 min prior to analysis.

### 4.4. Statistical Analysis

Data are expressed as means ± SEM. Statistical analysis was performed using Student *t* test by SPSS software (IBM). Statistical significance was defined as *p* < 0.05.
